# People’s Perspective on Out-of-Pocket Expenditure for Healthcare: A Qualitative Study From Pune, India

**DOI:** 10.7759/cureus.34670

**Published:** 2023-02-06

**Authors:** Deepu Palal, Sudhir L Jadhav, Shweta Gangurde, Kavita Thakur, Hetal Rathod, Johnson S, Prerna Verma, Sandeep Nallapu, Akhil Revikumar, Gayatri R Nair

**Affiliations:** 1 Community Medicine, Dr. D. Y. Patil Medical College Hospital and Research Centre, Pune, IND

**Keywords:** urban and rural communities, national health insurance reform, lack of health care provider knowledge, cost of hospitalization, cost of investigations, government and private sector, qualitative approach, government health scheme, lack of health insurance, out-of-pocket expenditure (oope)

## Abstract

Background

Out-Of-Pocket Expenditure (OOPE) directly reflects the burden of health expenses that households bear. Despite the availability of social security schemes providing healthcare benefits, a high proportion of Indian households are still incurring OOPE. In order to recognize the reasons behind OOPE, a comprehensive understanding of people's attitudes and behavior is needed.

Methodology

By purposive sampling, 16 in-depth interviews were conducted using an interview guide in the catchment area of urban and rural health centers of a tertiary healthcare hospital. Interviews were conducted in Marathi and Hindi and were audio tape-recorded after taking informed consent. The interviews were transcribed and translated into English, followed by a thematic analysis.

Results

Although most participants knew that government hospitals provide facilities and experienced doctors, inconvenience and unsatisfactory quality deter them from utilizing government facilities. A few had experiences with government schemes; almost all concur that the formality and procedure of claiming insurance are cumbersome and all have had bad experiences. Cost of medications and consultation accounted for the majority of the healthcare expenditures. While some participants had benefitted from insurance, few regretted not enrolling in one.

Conclusion

The awareness regarding government schemes was derisory. Government-financed health insurance schemes and their utilization are crucial to reducing OOPE. Efforts should be made to increase accessibility to public healthcare services. Nevertheless, there is potential to redress the barriers to improve scheme utilization.

## Introduction

According to the Organization for Economic Cooperation and Development, out-of-pocket expenditure (OOPE) is "any direct outlay by households, including gratuities and in-kind payments, to health practitioners and suppliers of pharmaceuticals, therapeutic appliances, and other goods and services whose primary intent is to contribute to the restoration or enhancement of the health status of individuals or population groups" [1]. OOPE on health refers to individuals' or families’ expenses for healthcare providers after deducting payment by insurance, government schemes, or hospital discounts. OOPE directly reflects the burden of health expenses that households bear. It includes expenditures on outpatient and inpatient services, antenatal, natal, and postnatal care, immunization, family planning, patient transportation, prescribed drugs, as well as other expenditures such as blood, oxygen, nutrition, etc., whether the contact was through referral or patient’s initiative [1-3]. According to National Sample Survey (NSS) Office's Consumer Expenditure Survey in 2011-12, 18% of households in India incurred "catastrophic" health expenditures [4]. According to the NSS 75th round, only 12.9% of the rural and 8.9% of the urban population is covered under government-sponsored insurance schemes, while the number of people not covered under any scheme remains at 85.9% and 80.9% in rural and urban areas, respectively [5]. OOPE creates havoc in people’s lives by having to decide between basic necessities such as food, shelter, clothing, and education and the life or health of loved ones. It often plunges citizens into the graveyard of poverty and eternal debts. Vasudevan et al. found that, in Puducherry, India, only 2.3% of households were covered by health insurance in rural areas while it was 1.7% in urban areas [6].

Numerous quantitative studies have been done to assess OOPE in general as well as for specific diseases. It is desirable to assess awareness, sources of information about government assistance and insurance schemes, and the reasons behind their non-utilization. Thakur et al., in Maharashtra, found in their study that despite being enrolled in the government scheme, several respondents had incurred OOPE due to a lack of awareness [7].

Despite the availability of social security schemes such as Mahatma Jyotirao Phule Jan Arogya Yojana (MJPJAY), Ayushman Bharat, Central Government Health Scheme (CGHS), Employment State Insurance Scheme (ESIS), and schemes like Pradhan Mantri Bhartiya Janaushadhi Pariyojana (PMBJP) providing healthcare benefits, a high proportion of Indian households are incurring OOPE. To recognize the reasons behind OOPE, a comprehensive understanding of people’s attitudes and behavior was needed. Hence, this study was planned.

## Materials and methods

By purposive sampling, 16 in-depth interviews were conducted using an interview guide in the field practice area of urban and rural health centers of a tertiary healthcare hospital, Dr. D.Y Patil Medical College, Pune, India. Interviews were conducted in Marathi and Hindi at the convenience of the participant at his or her residence. Interviews were audio tape-recorded after informed consent. The interviews were then transcribed and translated into English. Greetings and icebreakers were part of a formal introduction, followed by conversation. Each nonverbal indication, including gestures, nodding, and smiling was also recorded. The study participants were engaged in interviews up until there was no longer any new information to be obtained. Approximately 15-20 minutes were spent in each interview. The study participants were given opportunities to ask questions or clarify doubts after the in-depth interviews, and their concerns were answered. The notetakers provided notes that were verbatim accurate. Thematic analysis was done.

The study was approved by the Institutional Ethics Sub-Committee, Dr. D.Y. Patil Medical College, Hospital and Research Centre, Pune, India (Approval number: I.E.S.C/295/2021).

## Results

A total of 16 participants were interviewed, out of which five (31.25%) were males and 11 (68.75%) were females; 56.25% of the participants were from the urban area while 43.75% were from the rural area. The age of the participants ranged from 35 years to 60 years. The seven themes that emerged from the interviews were (i) cost related to health services, (ii) healthcare services utilization preferences: private vs public, (iii) understanding and utilizing public health schemes, (iv) viewpoint on health insurance, (v) expenditure on nutrition, (vi) coping strategies for health expenditure, (vii) and ideas and suggestions to improve OOPE. Participants are mentioned below as P1, P2, etc.

Cost related to health services

Most participants believed that the doctor's consultation fee was the most expensive, while a few believed that the investigations and medications were the most expensive.

I think most of the money is spent on medication. -P3

It is the medicine and specialist's fee which is most costly. -P5

More money is spent on medication, and doctor's fee is still manageable. -P9

Our concern is the fees (doctor's consultation) -P10

Healthcare services utilization preferences: private vs. public

Some participants were of the opinion that government hospitals had experienced doctors. Although most participants knew that government hospitals provide facilities, long waiting hours and concerns regarding the quality of treatment deter participants from utilizing government health facilities. Participants were of the opinion that they preferred private facilities even though they are expensive, due to the above factors.

I used a government hospital for vaccination during pregnancy. -P2

I used to go to a government hospital, but I don't prefer going now as it takes a lot of time. -P3

We prefer private hospitals over government hospitals as it takes a lot of time in government hospitals. -P5

Government hospitals are more advantageous too, as they have experienced doctors over there. -P6

I prefer going to private hospitals because we expect convenience from government hospitals, which we are not getting; although they are economically cheaper. The convenience I am talking about is in terms of formalities, regulations, and hospitality of a doctor. -P8

When you go to a government hospital it takes a lot of time, have to visit multiple times, and it is very much crowded; hence, I prefer to go to a private hospital. -P9

In government hospitals, they don't care much and results (treatment outcomes) are also delayed hence I go to private doctors.-P11

She further added that there are advantages to going to a government hospital; it is free of cost and there are good doctors too, but it is time-consuming.

We have to wait a long time for consultation in the government hospital. -P12

P12 took her ANC consultation from a private hospital but delivered the baby in a government hospital.

We want to use government hospitals for consultation and treatment but they ask us to come on a "specific day". -P 10

P10, a working couple, also said that in a government hospital, they fear how well a patient is taken care of.

I think government hospitals are good, there are qualified doctors over there, but still, I don't go to a government hospital. -P13

On being inquired about a reason for not visiting a government hospital, P13 responded, “Sometimes there are no facilities, sometimes facilities are not available on time."

Understanding and utilizing public health schemes

A few of the participants had used or had experience with government schemes; almost all concur that the formality and procedure of claiming insurance are cumbersome and they all have had bad experiences. The tedious process of documentation adds to the trouble of utilizing government schemes. Another participant said that despite knowing about the government scheme, he could not utilize it as he was a government employee. Contrastingly, a female participant took advantage of a government program that provided free transportation during her childbirth.

I am aware of government health schemes, but they are only used by a few people. I experienced it during my father's surgery when he met with an accident. The procedure to procure money was troublesome/inconvenient. It was time consuming to use the scheme under which I was enrolled. -P2

*I have used "Mahatma Phule Yojna" *(MJPJAY) *for my husband’s surgery (angioplasty); everything was included in the scheme. -P7*

I don't know the name of the scheme but I delivered my baby in a government hospital, and there was a free pick-up and drop service available at the hospital. -P12

I know about "Mahatma Phule Yojna" but since I am a government servant, it's not applicable to us; moreover, the scheme is not easily usable. -P13

Viewpoint on health insurance

Most participants agreed that health insurance has benefits and is essential in the event of hospitalization. A few of them regretted not having health insurance. One of the females responded that she had defaulted on payment and hence could not utilize the services during need. A female respondent said that she feels the hospital overcharges because of insurance, when they otherwise would not.

I had taken health insurance “three times” but did not develop any condition to use it, “so I defaulted on payment”. But then I got sick and couldn’t avail of any benefits. -P2

Health insurance has its benefits. -P5

I have used insurance, but I feel that if there is insurance, doctors charge more bill, they take more money. If there is no insurance, then the bill would have been less; it would have been around 20-25 thousand. -P11

P11 had given the above opinion on the use of insurance when her husband was admitted for dengue in a private hospital and was charged Rs. 45,000.

I had not taken insurance. Earlier I was yet to think about insurance. When I underwent surgery, I realized insurance is very important. -P13

Expenditure on nutrition

Only a few participants said that they had to spend extra cash on nutrition to maintain the health of the patient during illness. Expenses on nutrition cost a few hundred extras.

My husband had undergone cardiac surgery recently. We have spent more money on buying different oils for cooking, which is more costly than our regular oil. Even for my husband's weight management, we have to buy different food items, which costs us a lot. -P7

While my husband was admitted to the hospital for dengue, we had to spend more on fruit juices and fruits, which cost extra rupees 200-250 daily for nearly eight days. -P11

Coping strategies for health expenditure

Only two out of the 16 participants were short of money for their hospital expenses. Borrowing from friends was necessary for some to cope with the cost of hospitalization. In contrast, the majority refused to have taken debt for health expenses.

I have not borrowed money from anyone, but we take our medications from a known chemist and pay him the money in installments. -P7

I had to borrow money for my heart surgery. They were all my friends who had lent me money. At that time, I thought if I had taken health insurance, this situation of borrowing money from friends would not have arisen. Now I understand that Mediclaim (health insurance) is very important. -P13

Ideas and suggestions from participants to reduce OOPE

One participant suggested that the government should provide free medication for at least one person in the household if there are multiple patients with chronic diseases in a family. Another participant stated that her healthcare costs might have been lower if there were more generic drug outlets.

Generic medications are not available in shops near me; it's difficult to find a shop nearby. If more generic medicine shops were available, it would cut down on expenditure. -P6

We have multiple patients who have diabetes medicines for it. If the government makes a provision for free medication for at least one person, we will have to spend less on our end. -P7

I suggest that people who do not have insurance go to government hospitals. There they should be given good medications, and doctors should be more careful/ vigilant. -P11

I would suggest people to go to government hospitals -P12

P12 was a female participant, who had delivered her baby in a government hospital and availed of transportation facilities for the same.

We pay the same amount of tax as other countries, we are paying so much, we think the government should give us services. -P10

Another participant suggested that a kiosk of a public hospital should be made available closer to the community so that some documents related to government schemes and hospitalization could be processed faster or in advance.

## Discussion

Expenditure on healthcare is an important financial burden, which needs to be addressed. Catastrophic health expenditure (CHE) pushes millions into poverty every year. Poverty is a cause of poor health and vice-versa. Reducing OOPE and CHE helps in attaining Sustainable Development Goals 1 and 3.8 [8,9]. OOPE can affect different groups in different ways. Qualitative studies provide a deeper understanding of OOPE in healthcare. These could provide insight to policymakers to modify healthcare schemes and systems according to the needs of the people.

Costs related to health services

The visit to a doctor or hospital may increase a family's expenses. The expenditure involves a doctor’s consultation fee, medication, laboratory investigation, transport charges, and loss of pay. In our study, it was found that most patients reported that medications and doctor's fees accounted for the majority of their health expenditures.

Aji et al., in their research, discovered that all households with hospitalized family members were concerned about the additional costs of travel and meals for accompanying patients [10].

In a qualitative study by Liyange et al, they found that even though patients were admitted to a government facility, tests were sent to private laboratories. Some patients were taken care of by a hired caregiver. Patients' and relatives' travel to and from the health facility contributed to the costs. A few patients experienced a loss of income as a result of hospitalization, particularly those who were self-employed or held daily-paying jobs. If a family member who was taking care of the patient was also working, the family lost even more money [11].

Healthcare services utilization preferences: private vs. public

The availability of private hospitals in every nook and cranny of the country has made healthcare accessible for all. Despite government health services being free or at a nominal cost, the utilization of such facilities varies. Convenience and availability supersede the cost at the time of distress.

In this study, although most participants know that government hospitals provide exceptional facilities, long waiting hours and concerns regarding the quality of treatment were factors that deter participants from utilizing government health facilities. whereas a few advocated the advantages of having experienced professionals at government hospitals.

It is common to find facilities in the public sector providing substandard care [12]. Long wait times at government healthcare facilities is a significant factor driving the public to private healthcare facilities [13-16].

Understanding and utilizing public health schemes

Many government schemes are available to decrease OOPE, including free investigations, surgeries, medications, and transportation charges. MJPJAY is one scheme aiming to provide beneficiaries with high-quality, cashless healthcare for severe illnesses requiring hospitalization [17].

In this study, there is little awareness among people regarding government schemes. A few of them said that the formalities and procedures of claiming insurance (government and private) are cumbersome and have had bad experiences. A female participant utilized a government scheme that provided free transportation during childbirth. Another participant said that despite knowing about the government scheme, he could not utilize it because he was a government employee. The tedious process of documentation adds to the trouble of utilizing government schemes.

Findings from Govil et al. in their study from Rajasthan imply that Janani Suraksha Yojana (JSY) has improved institutional delivery coverage and decreased financial stress for households and families, but not enough for difficult deliveries [18]. Similarly in another study, subpar incentives, delayed payment, difficulty in arranging for residential proof, and a lot of administrative paperwork were some of the factors affecting a decreased acceptance of the scheme [19]. Other problems were OOPE even after enrolling in the program (43.2%), problems with comfort and care quality (32.4%), and delays in making use of the program (24.3%) [20].

Viewpoint on health insurance

Private and government health insurance utilization remains below that of other high- and middle-income countries; reasons could include a lack of knowledge, the high cost of the premium, or the belief that it is unnecessary.

Most of the patients in the current study believed that having health insurance had helped them, and a handful of them regretted not having insurance.

In another study, a participant mentioned that health insurance was a source of relief and helped cope with incurred costs from family members who had been hospitalized, while some participants reported lower satisfaction levels with their health insurance [9]. While in another study, some participants believed that if they did not get sick throughout the year, they would like to have their payments returned in full after the year [21].

Expenditure on nutrition

Good nutrition is essential for the upkeeping of good health. People tend to focus more on neglected nutrition when hospitalized or during an illness episode. Nutritious foods and supplements are expensive. Also, food for the caretaker during inpatient admission adds to the expense. Similar to this study, concern was also raised by a household whose members were hospitalized on the additional costs of travel and meals for accompanying patients [10].

Coping strategies for health expenditure

Money plays a crucial role in choosing the health facility to avail treatment. Private health facilities are associated with higher OOPE. Severe illness, prolonged hospitalization, major procedures, lack of insurance coverage, and low household income are some factors leading to impoverishment.

In the current study, borrowing from friends was necessary for some to cope with the cost of hospitalization. At the same time, the majority refused to have taken debt for health expenses. According to a study conducted by Mishra et al to meet the OOPE on institutional delivery, one in four mothers resorted to borrowing money or selling assets. High OOPE on delivery (institutional) necessitates the need to borrow money and sell assets [22]. While Sahu et al in Odisha found that only 15.7% borrowed money to cover OOPE [23].

Various coping mechanisms include spending savings funds, borrowing, selling assets, and receiving assistance from neighbors and relatives [10,24-26]. In a convergent parallel mixed methods study by Dalinjong et al. in North Ghana, participants explained how their families had liquidated assets to pay for the medications and ultrasound tests [27]. Receiving assistance from neighbors and relatives was the second most common way to address the financial cost [10,25,26]. A study by Balasubramanian et al. found 59% of patients used their savings, 7.8% paid via insurance, and 31.1% had to borrow money. A higher percentage of the poorest quintiles (47.2%) coped by borrowing money, while the majority (86.1%) of the wealthiest quintiles paid from their monthly income or savings or had health insurance [28].

Ideas and suggestions from participants to reduce OOPE

Some participants suggested that the government should provide free medication. Currently, there is no provision for free medications from pharmacies. However, under PMBJP, more Janaushadhi Kendras, which provide generic medications at 50-90% discount, are being set up to address these problems [29]. Also, public health facilities dispense free drugs based on consultation.

The availability of free drugs in government health facilities has declined from 31.2% to 8.9% for inpatient care and from 17.8% to 5.9% for outpatient care, and drugs constitute about 74% of private OOPE [30]. Inefficient drug procurement and management of the supply chain are linked with shortages of essential medications in government health facilities [31,32].

A participant commented that the expenditure would have been much lower if there were more generic drug outlets. Even though there are 8819 Janaushadhi Kendras spread across the country, they are not easily accessible compared to regular pharmacies [29]. State and cooperative society-run pharmacies also provide drugs at a discounted rate. Most participants were unaware of generic drug stores.

As discussed above, consultation at a government hospital involves long waiting and cumbersome procedures with almost no satisfaction. A few participants opined that the government should provide them with better services for their hefty tax.

Some of our study participants commented that a few doctors provide unsatisfactory services with steep consultation charges and in general, consultation fees could be reduced. Some participants believed that those who don't have health insurance should use government facilities more than private hospitals.

Another participant suggested that a kiosk of a public hospital should be made available closer to the community so that documents related to government schemes and hospitalization could be processed faster, in advance, or parallelly. It would save time and money for the beneficiaries and help to avoid repeated visits to procure documents. At the same time, It could also act as an enrollment center or information desk. This initiative may attract people to use public health facilities and schemes more as it may provide a rapid resolution to their concerns.

Limitations

The aim of the current study was to obtain the perspectives of the patients and associated factors regarding OOPE. However, for a comprehensive picture, the views and opinions of healthcare providers and other stakeholders should also have been taken into account to understand the constraints and impediments. The study also did not include the floating population. This qualitative study could not quantify OOPE since the sampling design precluded generalizing the findings.

## Conclusions

Medications and doctor's fees accounted for the majority of the health care expenditures. Having health insurance helps people in decreasing OOPE. People are not very aware of government programs and schemes. Inconvenience and concerns regarding the quality of treatment deter people from utilizing government health facilities. Awareness about government-financed health insurance schemes and their utilization is crucial to reducing OOPE. Increasing the number of government healthcare facilities in rural areas, flexible OPD timings, and availability of more doctors would directly contribute to reduced waiting time, which was found to be an important barrier to availing government healthcare facilities. Providing mobile healthcare services in remote and inaccessible areas would make primary healthcare attainable.

Nevertheless, there is potential to redress the barriers and improve scheme enrolment and utilization such as periodic enrolment campaigns in villages with prior notification, adding provision to panchayats or municipalities for enrolment and documentation support. The introduction of publicly funded health insurance for a broader audience may significantly help ease economic hardship for families as the majority of the economic risk from ill health appears to be tied to OOPE.
